# *A. castellanii* and *P. aeruginosa* mutually exacerbate damage to corneal cells during coinfection

**DOI:** 10.1128/spectrum.02683-23

**Published:** 2023-12-14

**Authors:** Chun-Hsien Chen, Chen-Chieh Liao, Yu-Jen Wang, Fu-Chin Huang, Wei-Chen Lin

**Affiliations:** 1 Department of Parasitology, College of Medicine, National Cheng Kung University, Tainan, Taiwan; 2 Department of Physiology, College of Medicine, National Cheng Kung University, Tainan, Taiwan; 3 Department of Parasitology, School of Medicine, China Medical University, Taichung, Taiwan; 4 Department of Ophthalmology, College of Medicine, National Cheng Kung University Hospital, National Cheng Kung University, Tainan, Taiwan; 5 Department of Microbiology and Immunology, College of Medicine, National Cheng Kung University, Tainan, Taiwan; 6 Institute of Basic Medical Sciences, College of Medicine, National Cheng Kung University, Tainan, Taiwan; University of Huddersfield, Huddersfield, United Kingdom

**Keywords:** *Acanthamoeba castellanii*, *Pseudomonas aeruginosa*, corneal cells, coinfection

## Abstract

Microorganisms that are involved in microbial invasion keratitis infiltrate the cornea and cause eye pain. Despite the administration of antimicrobial treatment, patients with amoebic keratitis develop bacterial coinfections. *Pseudomonas aeruginosa* infection accounts for 50% of the reported bacterial coinfections in these patients. However, the role of pathogenic bacteria in amoeba-induced corneal damage remains unclear. In our current study, we conducted assays to examine cytopathic effects and observed increased corneal cell damage in the group with secondary *P. aeruginosa* infections. Imaging revealed that the presence of *A. castellanii* enhanced the accumulation of *P. aeruginosa* in areas of cell monolayer leakage. We demonstrated that treatment of *P. aeruginosa* with amoeba-soluble antigen resulted in higher adhesion ability, increased biofilm formation, and more severe corneal cell damage. Overall, this research significantly contributes to our understanding of the risk of *P. aeruginosa* coinfection in the progression of Acanthamoeba keratitis.

**IMPORTANCE:**

At the National Cheng Kung University Hospital, numerous cases of amoebic keratitis had been identified with concurrent bacterial infections. Among these bacterial coinfections, *Pseudomonas aeruginosa* accounted for 50% of the reported cases. However, the impact of pathogenic bacteria on amoeba-induced corneal damage remains unclear. In our study, we successfully demonstrated that *P. aeruginosa* accumulated on the *Acanthamoeba castellanii* surface and caused more severe corneal damage. We also indicated that the exposure of *P. aeruginosa* to amoeba-soluble antigens enhanced its adhesion ability, promoted biofilm formation, and led to more severe corneal cell damage. These findings significantly contributed to our understanding of the risk associated with *P. aeruginosa* coinfection in the progression of amoeba keratitis.

## INTRODUCTION

*Acanthamoeba castellanii* is a genus of free-living amoebae, and its members are found in soil, water, and other environments worldwide (1). Although other genotypes, such as T2, T3, T5, T6, T8, T9, T11, T13, and T15, have also been identified in Acanthamoeba keratitis (AK) patients, clinical studies have shown that the most prevalent pathogenic genotype in both keratitis patient samples and environmental samples is T4 (2). The T4 genotype is associated with high invasiveness and severe cytotoxicity to host cells (2). *A. castellanii* infections typically occur in the eyes, skin, or central nervous system (3). AK causes symptoms including eye pain, redness, blurred vision, and blindness (4). The development of *A. castellanii* infections is often attributed to risk factors, such as the use of contact lenses and exposure to contaminated water sources (4). Treatment of amoeba infections typically comprises a combination of antimicrobial agents, including polyhexamethylene biguanide and chlorhexidine (5, 6). However, the failure of topical treatments can occur due to the formation of amoebic cysts and disruption of the environment in the eye. Surgical options, including corneal transplantation, are necessary in the majority of AK cases to control the disease (7).

In addition to protozoan infections, microbial invasion keratitis develops via a process whereby microorganisms, such as bacteria and fungi, infiltrate the cornea, leading to infection (8). Disruption of the corneal epithelial barrier provides an entry point for microorganisms, triggering inflammation and damaging tissue (9). *Pseudomonas aeruginosa* is an opportunistic pathogen that is commonly found in a wide range of environmental habitats, including soil, water, plants, and animals (10). *P. aeruginosa* has been demonstrated to be associated with a range of infections, including urinary tract infections, pneumonia, wound infections, and sepsis (11). Corneal infection is one of the prominent clinical manifestations associated with *P. aeruginosa* infections and is often attributed to factors such as contact lens use or eye injury (12). *P. aeruginosa* species are the organisms that are most frequently isolated from patients with microbial keratitis at the National Taiwan University Hospital (13). Records from microbial keratitis patients in the Mid-Atlantic Region of the United States reveal that the most common Gram-negative isolate among culture-positive bacterial keratitis cases was *P. aeruginosa* (12). Symptoms of *P. aeruginosa* corneal infection include severe eye pain and redness. *P. aeruginosa* produces a variety of virulence factors, such as exotoxins, proteases, and lipopolysaccharides, which allow it to colonize and invade host tissues (8, 14, 15). Biofilms are a critical factor in the pathogenesis of *P. aeruginosa* infections, as they provide protection from host immune responses and antimicrobial agents (16, 17). The biofilm lifestyle of *P. aeruginosa* also contributes to its persistence in clinical settings, where it can survive on surfaces and medical devices (18). Treatment of *P. aeruginosa* corneal infection typically involves a combination of topical and systemic antibiotics such as fluoroquinolones, aminoglycosides, and cephalosporins (12).

Despite the administration of a combination of antimicrobial agents, patients with amoebic keratitis at the National Cheng Kung University Hospital were found to have bacterial infections. Furthermore, clinical AK cases with bacterial coinfections were associated with more severe ocular symptoms. Among the reported AK cases of bacterial coinfection from National Cheng Kung University Hospital, *P. aeruginosa* infection accounted for 50% of the bacterial infections identified. *A. castellanii* and *P. aeruginosa* are widespread pathogenic microorganisms that cause certain human diseases (18, 19). Experimental evidence suggests that *A. castellanii* can interfere with the efficacy of hospital disinfection with chlorine, which leads to increased bacterial survival (11). Animal studies have indicated that bacterial presence participates in the development of AK (20). Additionally, previous studies have shown that coculturing bacteria such as *P. aeruginosa* spp. and *Legionella* spp. with amoebae can cause the bacterium to transform into an active and virulent form (21, 22). On the other hand, it is interesting that a past study indicated that the presence of normal ocular flora reduces amoeba-induced corneal damage (23). Therefore, the role of pathogenic bacteria in amoeba-induced corneal damage remains unclear. This study aimed to investigate the impact of *P. aeruginosa* on *A. castellanii* infections. Through assays to measure cytopathic effects, we observed increased accumulation of *P. aeruginosa* on corneal cell leakage sides and more corneal cell damage in the group with secondary *P. aeruginosa* infections. In addition, we demonstrated that treatment of *P. aeruginosa* with amoeba-soluble antigen led to higher adhesion ability, faster biofilm formation, and more severe corneal cell damage. This study sheds light on the complex interactions between bacteria and amoebae, providing valuable insights that can potentially guide the development of effective treatment strategies for this vision-threatening condition.

## RESULTS

### *P. aeruginosa* induced severe secondary infections

**Fig 1:**
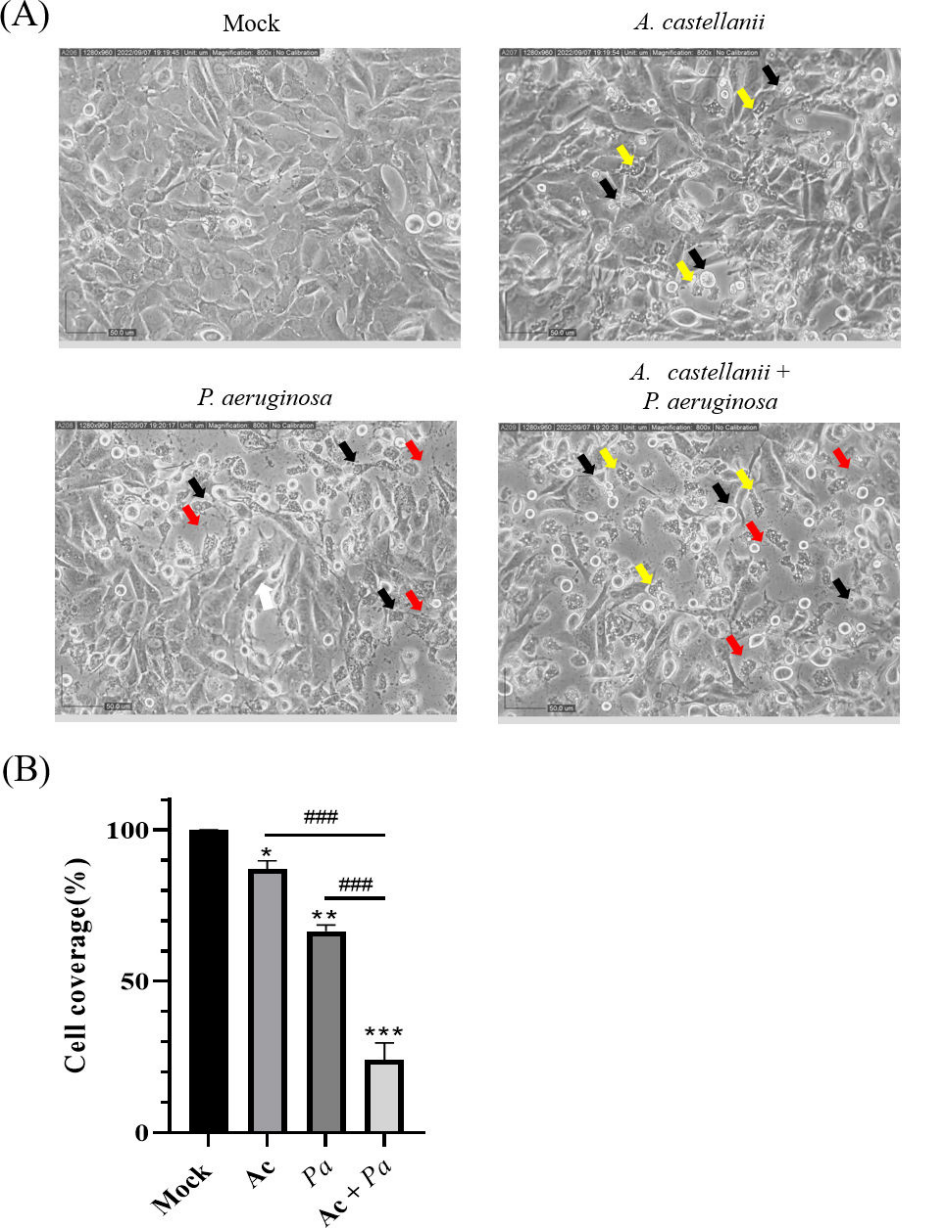
*A. castellanii* and *P. aeruginosa* exerted cytopathic effects on SIRC cells. (**A**) Monolayers of SIRC cells were coincubated with or without *A. castellanii* for 2 hours, and then the cells were incubated with or without *P. aeruginosa* for 1 hour. The cell coverage was assessed using a microscope (yellow arrow: *A. castellanii*, red arrow: *P. aeruginosa*, and black arrow: rounding or damaged SIRC cell). (**B**) Pathogen-induced cell damage was quantified by attached SIRC cell coverage. Cell coverage was revealed by ImageJ analysis. *Ac*, *A. castellanii* and *Pa*, *P. aeruginosa* (*P < 0.05, **P < 0.01, and ***P < 0.001 compared with the mock group; ###*P* < 0.001 compared with the secondary bacterial infection group).

In previous studies, *P. aeruginosa* infections have been reported in almost two-thirds of patients with contact lens-associated microbial keratitis (13). Additionally, more severe keratitis symptoms are found in amoeba keratitis patients with secondary bacterial infections. Therefore, to evaluate whether secondary bacterial infections cause severe corneal damage, we established an *in vitro* model of secondary infection. In the secondary infection group, *P. aeruginosa* was added to the culture environment after the amoeba had invaded the confluent monolayer of rabbit corneal cells. As indicated by the black arrows in Fig. 1, shrinkage and rounding of Statens Seruminstitut Rabbit Cornea (SIRC) cells were observed in the presence of amoebae. Bacterial infection caused the rounding of SIRC cells and induced cell lysis. As shown in the secondary infection group, the bacteria-induced cell lysis near the area of the cell monolayer with amoeba-induced damage. The results showed that secondary bacterial infection induced a more significant cytopathic effect than those observed in the other single pathogen infection groups. According to the *in vitro* assay, our study demonstrates that secondary *P. aeruginosa* infection leads to significantly severe corneal cell damage.

### *P. aeruginosa* adhered to the surface of pathogenic *A. castellanii*

**Fig 2:**
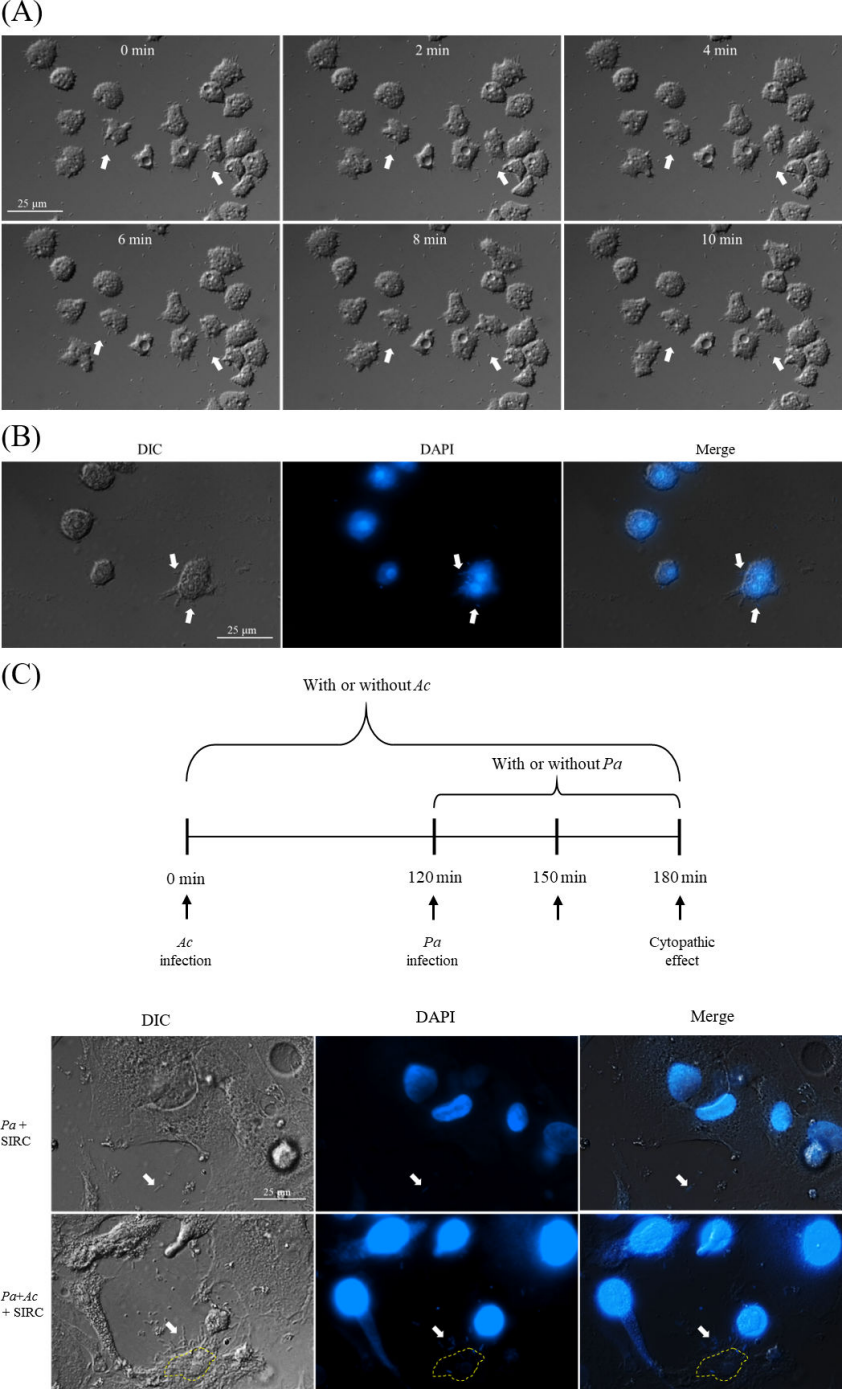
*P. aeruginosa* accumulated on the surface of *A. castellanii*. (**A**) Time-lapse microscopy was utilized to observe the coincubation of *A. castellanii* and *P. aeruginosa*, with observations made every 2 minutes for a total duration of 10 minutes. (**B**) DAPI staining also revealed bacterial accumulation on the surface of *A. castellanii* at 10 minutes. (**C**) SIRC cells were treated with or without *A. castellanii*. After bacterial infection for 30 minutes, DAPI was used to stain the SIRC cells. *Ac*, *A. castellanii* and *Pa*, *P. aeruginosa* (white arrow: *P. aeruginosa* and yellow dotted line: *A. castellanii*).

Previous research suggests that *P. aeruginosa* can sense amoebae and attack them by adhering to the amoeba surface (19, 24). The images in Fig. 1 revealed that the bacteria seemed to accumulate near *A. castellanii* that had attached to areas of damaged cell monolayers. Based on the previous results, we suggested that *P. aeruginosa* might sense the presence of amoebae that had attached to corneal cells, increasing corneal cell damage. To reveal whether *P. aeruginosa* could accumulate around pathogenic *A. castellanii*, we conducted fluorescence staining. Over time, the time-lapse images revealed a gradual accumulation of *P. aeruginosa* surrounding *A. castellanii*. After 30 minutes of coincubation, the images indicated that *P. aeruginosa* accumulated on the surface of *A. castellanii* (Fig. 2A and B). According to a previous study, we suggested that the fluorescence results were caused by bacterial attachment, not amoeba phagocytosis (24). In addition to adhering to amoebae, *P. aeruginosa* invasion starts with sensing and adhering to host cells (25, 26). In the context of multiple infections, the adhesive preference of *P. aeruginosa* remains unclear. Therefore, we conducted further investigations to determine the adhesive preference of *P. aeruginosa* under conditions of secondary infection. Under the microscope, initial fluorescence was observed as bacteria aggregated on the amoebae that had already attached to corneal cells. We also observed that bacteria attached to the amoeba cell perimeters (Fig. 2C). Thus, based on these results, we suggested that the ability of *P. aeruginosa* to sense amoebae might be implicated in the development of severe secondary infections.

### Evaluation of the impact of the amoeba-soluble antigen on the growth of *P. aeruginosa*

**Fig 3:**
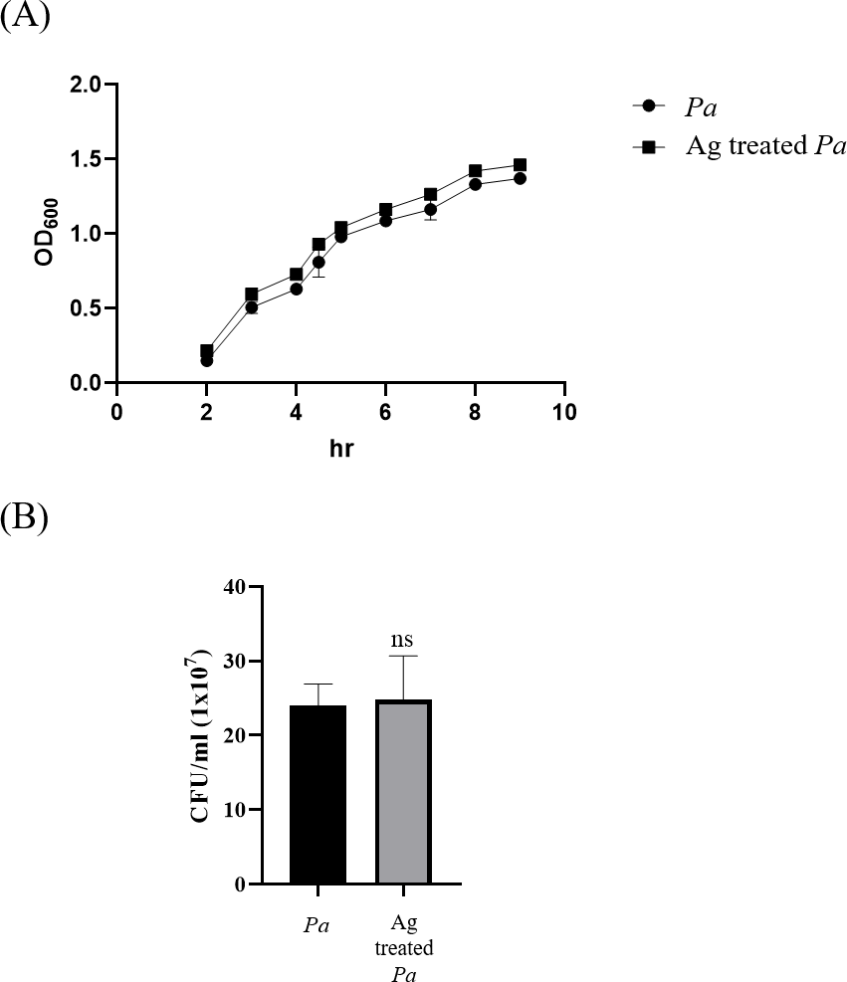
The growth of *P. aeruginosa* with and without amoeba-soluble antigens. (**A**) The growth curve of *P. aeruginosa* with and without amoeba-soluble antigens (Ag) (50 µg/mL) was evaluated by measuring the OD_600_ every hour until the bacteria reached the stationary stage of growth. (**B**) After 3 hours of incubation, the cell numbers of *P. aeruginosa* with and without amoeba-soluble antigens (Ag) (50 µg/mL) were measured by colony-forming unit (CFU) assay. *Pa*, *P. aeruginosa* and Ag, amoeba-soluble antigens.

The potential cytotoxicity of *A. castellanii* proteases has been reported in previous studies (27, 28). Bacteria might interact with soluble amoeba antigens within the corneal surface. However, no study, to the best of our knowledge, has investigated the effect of amoeba-soluble antigens on *P. aeruginosa*. Therefore, we further aimed to reveal the effect of amoeba-soluble antigens on *P. aeruginosa*. First, we assessed the effect of amoeba-soluble antigens on bacterial growth. OD_600_ measurements were used to evaluate the impact of amoeba-soluble antigens on the bacterial growth curve. Our results showed that the addition of amoeba-soluble antigens had no influence on the OD_600_ measurements (Fig. 3A). To confirm the effect of amoeba antigens, we estimated the number of viable bacteria at the exponential phase and stationary phase. The results revealed that the addition of amoeba-soluble antigens had no significant impact on colony units formed compared to the normal culture group (Fig. 3B). The results indicated that amoeba-soluble antigens had no impact on the growth of *P. aeruginosa*.

### Evaluation of the pathogenicity of amoeba-soluble antigen-treated *P. aeruginosa*

**Fig 4:**
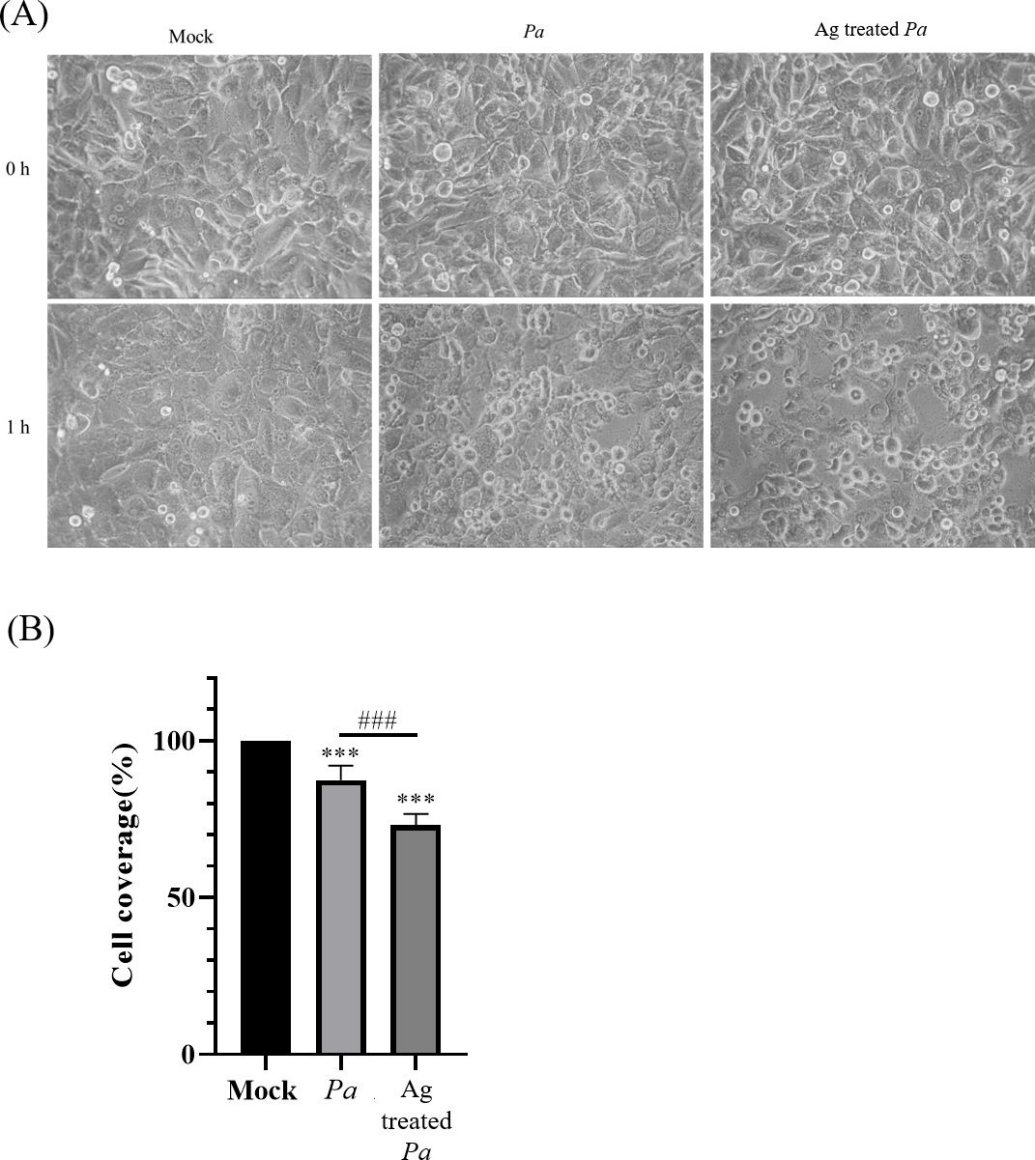
The increased pathogenicity of amoeba-soluble antigen-treated *P. aeruginosa* in SIRC cell culture. After *P. aeruginosa* was incubated with or without amoeba-soluble antigen, the bacteria were resuspended in Dulbecco’s Modified Eagle Medium and added to monolayers of SIRC cells. (**A**) Images were captured using a microscope at 1 hour. Mock: cell-only group. (**B**) After *P. aeruginosa* was infected for 1 hour, pathogen-induced cell damage was quantified by attached SIRC cell coverage. Cell coverage was revealed by ImageJ analysis. *Pa*, *P. aeruginosa* and Ag, amoeba-soluble antigens (***P < 0.001 compared with the mock group; ###*P* < 0.01 compared with *Pa* group).

A previous study revealed that long-term coculture with living *A. castellanii* decreases the pathogenicity of *P. aeruginosa* (19). The impact of amoeba-soluble antigens on the pathogenicity of *P. aeruginosa* remains unclear. To reveal the cytotoxic effect of amoeba antigen-treated bacteria, we conducted an assay to analyze cytopathic effects. The figure showed that bacterial infection caused corneal cell shedding and rounding, and the amoeba-soluble antigen-treated bacteria increased cell shedding (Fig. 4). The results showed that the growth of amoeba-soluble antigens incubated *P. aeruginosa* caused a significantly higher cytopathic effect on corneal cells than the normal culture condition. Here, we first demonstrated that amoeba-soluble antigens increased the cytotoxic effects of *P. aeruginosa* on cornea cells.

### Evaluation of the attachment of amoeba-soluble antigen-treated *P. aeruginosa*

**Fig 5:**
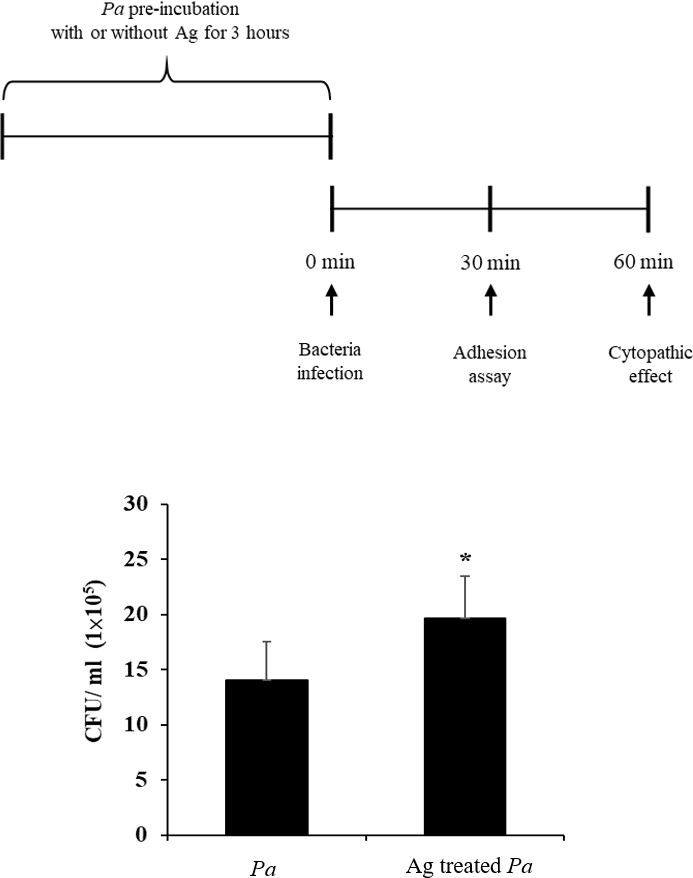
The adhesion of treated *P. aeruginosa* to SIRC cells. After incubation with or without amoeba-soluble antigens for 3 hours, *P. aeruginosa* was resuspended in Dulbecco’s Modified Eagle Medium and added to monolayers of SIRC cells. After a 30-minute bacterial invasion, adherent *P. aeruginosa* to SIRC cells was quantified by CFU counting assays. [*Pa*, *P. aeruginosa* and Ag-treated *Pa*, *P. aeruginosa* incubated with amoeba-soluble antigens (50 µg/mL)] (*P < 0.05, compared to *Pa* group).

Successful adhesion is the first step of bacterial pathogen infection (25). Due to the increased cytotoxic effect induced by the addition of amoeba-soluble lysates, we investigated whether preincubation with amoeba-soluble antigens could enhance bacterial adhesion. Through colony-forming unit (CFU) counting, the results of the adhesion assay showed that the addition of amoeba-soluble antigens significantly increased the adhesion of *P. aeruginosa* to corneal cells compared to the normal culture condition (Fig. 5). Thus, our results indicated that pre-incubation with amoeba-soluble antigens enhanced the attachment of *P. aeruginosa* to corneal cells.

### Evaluation of biofilm formation of amoeba-soluble antigen-treated *P. aeruginosa*

**Fig 6:**
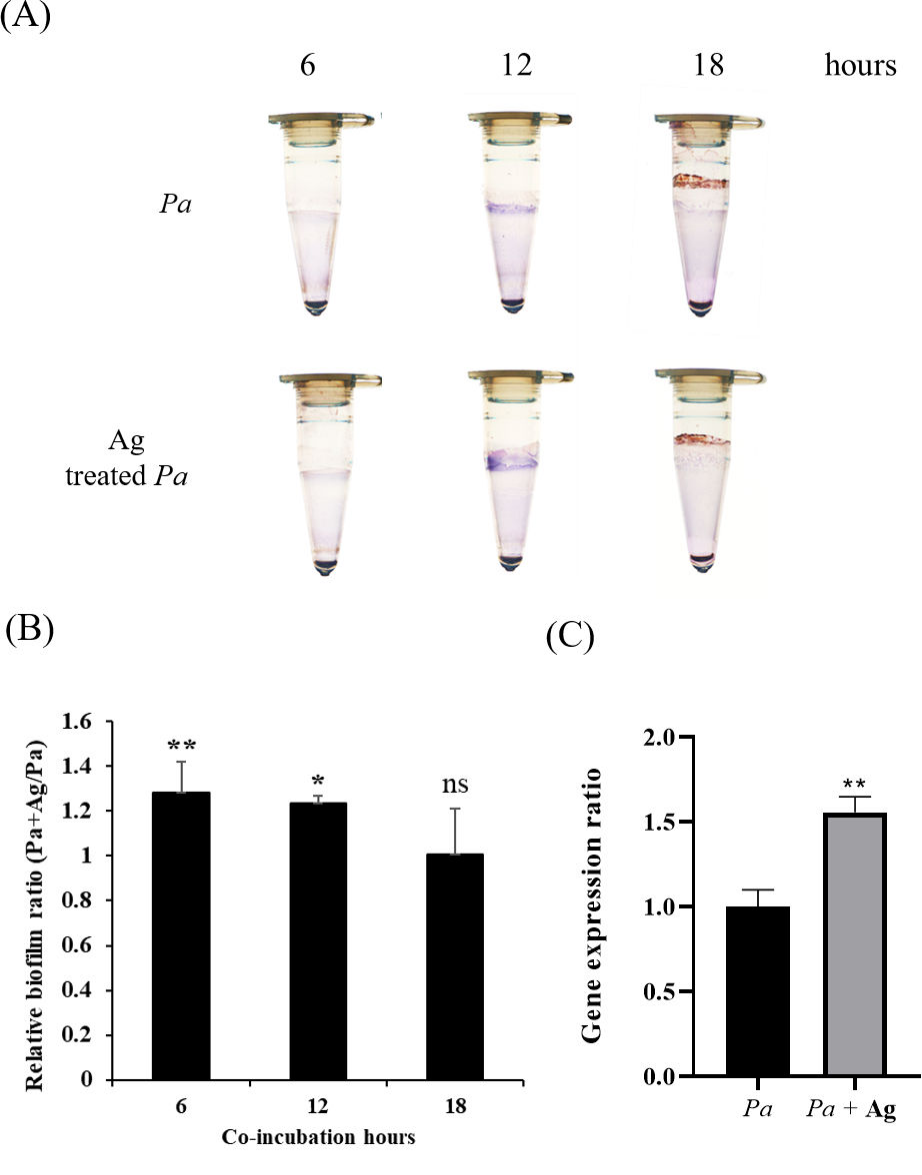
Biofilm formation of amoeba-soluble antigen-treated *P. aeruginosa*. The biofilm mass was taken. (**A**) Images by biofilm staining [*Pa*, *P. aeruginosa*; *Pa* + Ag, *P. aeruginosa* incubated with amoeba-soluble antigens (50 µg/mL)]. (**B**) The relative ratio of biofilm mass was quantified by the OD_550_ measurements at 6, 12, and 18 hours. (**C**) After 6 hours of incubation with or without amoeba-soluble antigen, the expression of *algD* in *P. aeruginosa* was measured by qPCR analysis (*P < 0.05 and **P < 0.01, compared to *Pa* group).

The virulence factors of *P. aeruginosa* include biofilm formation, secretion systems, and toxins (15, 16). Figure 5 reveals the increased attachment of bacteria after the addition of amoeba-soluble antigens. Bacterial attachment is a crucial process that enables bacteria to adhere to surfaces and form complex communities known as biofilms (29). To measure biofilm formation, the biomass of *P. aeruginosa* biofilms was stained with crystal violet at 6, 12, and 18 hours of growth in each group. The figure shows that the addition of amoeba-soluble antigens at the beginning of bacterial growth resulted in greater biofilm mass at 6 hours (Fig. 6). There was no significant difference in biofilm formation between the two groups at 18 hours. The alginate biosynthesis gene *algD* is an important gene for *P. aeruginosa* biofilm formation (16, 30). The expression levels of *algD* were increased in the amoeba-soluble antigen groups compared to the normal group at 6 hours. The epithelial cell lysates can enhance the expression of *P. aeruginosa* toxin, *ExoS*, by upregulating the type three secretion system (18). Our results showed that amoeba-soluble antigens did not impact the expression of the effector proteins of the *P. aeruginosa* type III secretion system, *ExoU*, *ExoT*, and *ExoY* (S1). Our results demonstrated that the exposure of *P. aeruginosa* to the amoeba-soluble antigens induced the development of biofilm formation compared to the normal culture group.

## DISCUSSION

*P. aeruginosa* and AK are highly correlated with the use of contact lenses (12). Additionally, inappropriate contact lens use or exposure to microorganism-contaminated water sources may lead to microbial infections, such as infections with *A. castellanii* and *P. aeruginosa* (3, 10). The records from National Cheng Kung University Hospital indicate that bacterial coinfection occurs in AK patients. Among the patients with coinfections, *P. aeruginosa*, which is the organism that is most commonly isolated from hospital patients with microbial keratitis, causes 50% of bacterial infections and leads to more severe corneal syndromes than amoeba keratitis. Our results demonstrate that secondary bacterial infection causes significant cell lysis and increased cell damage compared with single pathogen infections. According to previous evidence, the presence of bacteria decreased the effect of amoeba-targeting drugs and increased the survival rate of amoeba (6). Studies have revealed that *A. castellanii* can interfere with the bactericidal effect of antibiotics and improve the rate of *P. aeruginosa* survival after exposure to adequate chlorine disinfection (11). Therefore, we suggest that antibiotics and anti-amoeba cocktail therapies should be used to improve the treatment of AK.

Secondary infection with *P. aeruginosa* caused significant cell damage compared with single pathogen infection. The subsequent fluorescence results revealed that *P. aeruginosa* accumulated on the surface of pathogenic *A. castellanii* that had attached to corneal cells. *P. aeruginosa* was previously shown to attack amoebae and cells by attaching to the surface and injecting the cells with virulent substances (19). *P. aeruginosa* isolates that had not adapted to amoebae exhibited chemotaxis and rapidly swam toward the amoebae (19). A study revealed that coordination of the *P. aeruginosa* attack against *A. castellanii* relies on the use of the *P. aeruginosa* quinolone signal (24). Based on the available evidence, we propose that the accumulation of *P. aeruginosa* on amoeba cells amplifies bacterial attachment to the cell barrier that was damaged by amoebae. Consequently, the increased recruitment of bacteria contributes to the exacerbation of secondary barrier damage. Here, we suggest that *A. castellanii* may be considered a guide that facilitates bacterial accumulation at the invasion area, while *P. aeruginosa* contaminates the corneal surface of AK patients.

Previous amoeba-bacteria studies emphasize the interaction between the living amoeba and bacteria to investigate the underlying mechanisms (11, 18, 22). *P. aeruginosa* exists in a viable but not culturable state, which makes the bacteria highly resistant to antibiotic treatment but confers low virulence (31). Culture in the presence of amoebae can lead to a return of the viable but not culturable state of *P. aeruginosa* (21). Additionally, researchers have indicated that bacterial resuscitation might occur in piped water that contains amoeba (21). As *A. castellanii* and *P. aeruginosa* are widespread microorganisms, it has been reported that *A. castellanii* contributes to the colonization of adequate chlorine disinfection water systems by *P. aeruginosa* (11). Long-term coevolution of *P. aeruginosa* with *A. castellanii* leads to reduced bacterial virulence in *Caenorhabditis elegans* (19). Past studies indicated that the cell lysate of *A. castellanii* containing various amoeba proteases exerted cytotoxic effects on host cells (27, 28). No study has investigated the effect of amoeba-soluble antigens on *P. aeruginosa*. Our results indicated that short-term incubation with amoeba-soluble antigens enhanced the attachment of *P. aeruginosa* to corneal cells and caused significant cell damage compared with the normal culture group. Moreover, the results were substantiated by the biofilm assay, demonstrating that *P. aeruginosa* incubated with amoeba-soluble antigen exhibited accelerated biofilm formation, which may correlate with enhanced adhesion capability. An alginate-overproducing *P. aeruginosa* strain could defend against protozoan grazing in the early stage of microcolony formation (18). Through the upregulation of the type three secretion system, *P. aeruginosa* toxin (*ExoS*) expression and secretion can be induced by incubation with epithelium cell lysates (18). In this study, incubation with amoeba-soluble antigens did not affect toxin expression; on the other hand, amoeba-soluble antigens upregulated the expression of biofilm formation-related genes. Collectively, we suggest that amoeba-soluble antigen not only causes cell detachment but also increases the chances of bacterial infection in the amoeba-invaded corneal environment.

Protozoan-bacteria multiple pathogenic infections influence the host in various ways (32). Protozoan infection can modulate host immune responses and create a favorable environment for the growth of other pathogens, leading to harmful bacterial invasion (33). *Trichomonas vaginalis* decreases Lactobacillus colonization and upregulates proinflammatory responses. Changes in the vaginal environment enhance the growth of vaginosis-associated bacteria, such as *Gardnerella vaginalis,* and amplify the inflammatory response (34). Additionally, some pathogens can modulate the expression of their virulence genes during their interactions with other pathogens (33). Phagocytosis of enteropathogenic *Escherichia coli* bacteria strains augments the cytopathic effect of *Entamoeba histolytica,* upregulates the expression of Gal/GalNAc lectin on the amoebic surface, and enhances amoeba cysteine proteinase activity (35). Our results indicated that *P. aeruginosa* accumulated on the surface of *A. castellanii* and that this secondary infection augmented the cytopathic effect on corneal cells. Additionally, amoeba-soluble antigens enhanced bacterial biofilm formation and bacterial cytotoxicity. Based on our results, we suggest that the interaction of protozoa and bacteria may play an important role in AK progression. This is the first study to reveal the corneal cytopathic effect of AK-associated pathogen interactions.

### Conclusions

**Fig 7:**
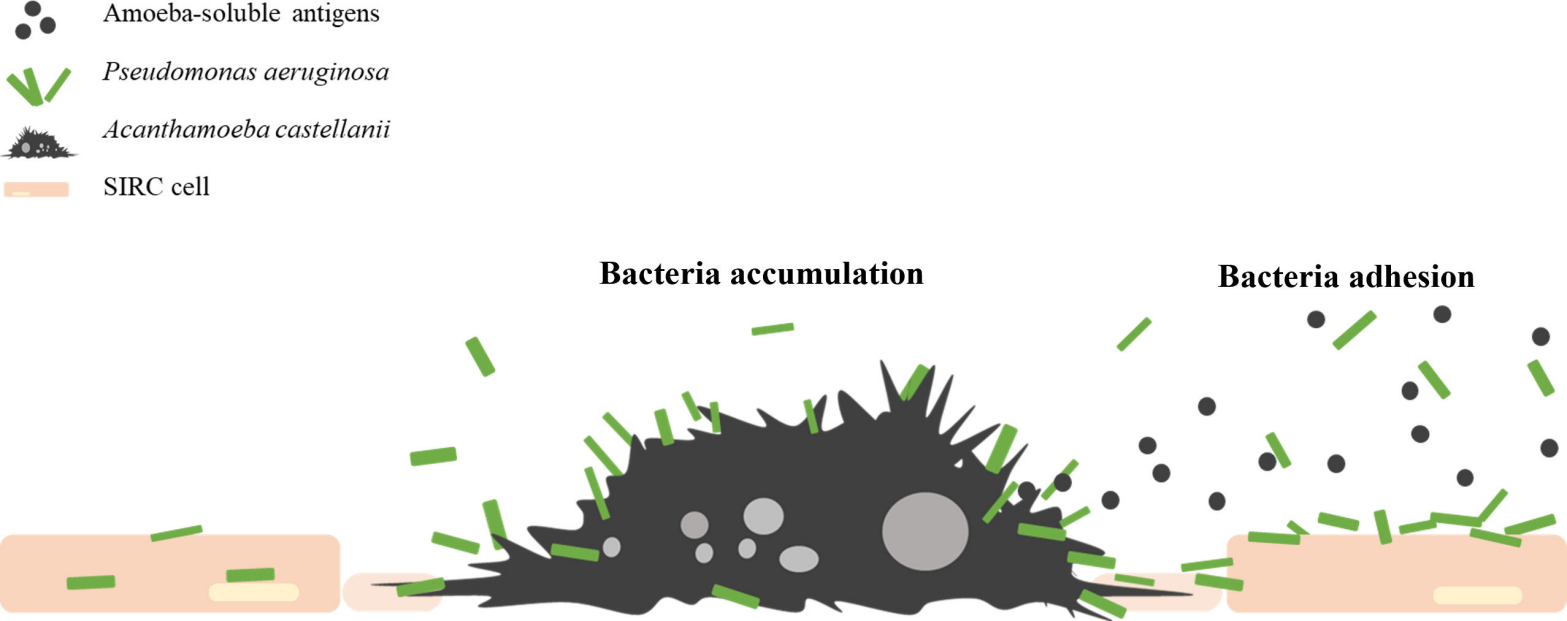
The potential role of *P. aeruginosa* in *A. castellanii* infection. *P. aeruginosa* increasingly attached to *A. castellanii* and caused greater corneal cell damage. The incubation with amoeba-soluble antigen-treated *P. aeruginosa* resulted in greater cell damage, more bacterial adhesion, and faster biofilm formation.

Our results demonstrated that secondary *P. aeruginosa* infection resulted in more severe cell damage, and we also observed the accumulative preference of the bacteria on *A. castellanii* cells that had attached to areas of monolayer leakage. Additionally, we demonstrated that the presence of amoeba-soluble antigen enhanced *P. aeruginosa* adhesion, biofilm formation, and cell damage (Fig. 7). Overall, our findings highlight the potential role of *P. aeruginosa* infection in the risk of AK progression, offering new insights into microbial keratitis.

## MATERIALS AND METHODS

### *A. castellanii* culture and amoeba-soluble lysate preparation

The pathogenic strain of *A. castellanii* used in this study, ATCC_50370 genotype T4, was obtained from the American Type Culture Collection. *A. castellanii* was cultured in proteose peptone-yeast extract-glucose medium in a T75 flask at 28°C. The amoeba cells were subsequently washed with Page’s modified Neff’s amoeba saline (PAS) and passaged until they reached a confluence of 80%. To prepare amoeba soluble protein, supernatants of cultures were removed, and adherent trophozoites were gently washed twice with PAS. Amoeba cells were then detached by 15-min incubation with PAS in an ice bath, transferred to polypropylene tubes, and resuspended in phosphate-buffered saline. Amoeba cells were lysed by using ultrasonic treatment (BO3 ultrasonic processor UP 1200; Chrom Tech, India) and then centrifuged at 16,000 × *g* for 15 minutes at 4°C to remove the insoluble pellets. The protein concentrations were measured by using a protein assay (Bio-Rad, USA) (6).

### Bacterial culture

*P. aeruginosa* PA14 (provided by Dr. Chang-Shi Chen, Department of Biochemistry and Molecular Biology, College of Medicine, National Cheng Kung University, Tainan, Taiwan) was cultured overnight with lysogeny broth (LB) on a rotary shaker (170 rpm) at 37°C.

### Cell culture

SIRC cells, obtained from the Bioresource Collection and Research Center, were cultured in Dulbecco’s Modified Eagle Medium (DMEM) supplemented with 10% heat-inactivated fetal bovine serum and 1% penicillin-streptomycin. The cells were maintained as monolayers at 37°C and 5% CO_2_ in routine culture. For the subsequent cytopathic assay, cell monolayers were prepared by overnight cell culture. Subsequently, the old medium was replaced with serum- and antibiotic-free DMEM.

### Cytopathic effect analysis

SIRC cells were cultured in 24-well plates as confluent monolayers. Before treatment, the overnight culture medium was replaced with serum-free DMEM. For the secondary infection assay, cell monolayers were incubated with the same volume of serum-free DMEM or *A. castellanii* ATCC_50370 (10^5^ amoeba cells/well) for 2 hours at 37°C and 5% CO_2_, and then the same volume of serum- and antibiotic-free DMEM or *P. aeruginosa* cells resuspended in serum- and antibiotic-free DMEM (4 × 10^6^ CFU/well) was added to the wells. After bacterial infection for 1 hour at 37°C and 5% CO_2_, the cell layers of all the groups were fixed with 2% paraformaldehyde, and the area of cell attachment was quantified by using an Olympus microscope (Tokyo, Japan) with a digital camera (AM7025X Edge; Dino-Lite, Taiwan) to evaluate the cytopathic effect of pathogens. The cell-damaged area was quantified in each treatment well of plates by ImageJ (National Institutes of Health, Bethesda, MD, USA).

To measure the cytopathic effect of the amoeba-soluble antigens-treated *P. aeruginosa*, bacteria were incubated with or without amoeba-soluble antigen (50 µg/mL) lysogeny broth on a rotary shaker (170 rpm) at 37°C for 3 hours. Then, 4 × 10^6^ CFU/well bacteria, which were centrifuged and resuspended in serum- and antibiotic-free DMEM, were added to the prepared SIRC cell monolayers. After bacterial infection for 1 hour, the cells were fixed with 2% paraformaldehyde, and the cytopathic effect of the pathogens was quantified by using a microscope. The cell-damaged area was quantified in each treatment well of plates by ImageJ.

### Microscopic analysis and CFU counting of *P. aeruginosa* accumulation and adhesion

To observe bacterial adhesive preference in the secondary infection model, corneal cells were seeded overnight to form a monolayer. Corneal monolayers were treated with or without amoeba cells (10^5^ amoeba cells/well) in serum- and antibiotic-free DMEM at 37°C and 5% CO_2_. After amoeba invasion for 2 hours at 37°C and 5% CO_2_, *P. aeruginosa* (4 × 10^6^ CFU/well) was added to the dishes. DAPI accumulates in the nuclei's DNA and is a commonly used nuclear counterstain in fluorescence microscopy. For fluorescence images, dishes were fixed with 2% paraformaldehyde and then stained with FluoroShield containing DAPI (4′,6-diamidino-2-phenylindole) (Ab104139; Abcam) after bacterial infection for 30 minutes. Then, we used an Olympus MV PLAPO 2XC dissection microscope (Tokyo, Japan) to measure the image.

To observe bacterial adhesion on *A. castellanii*, amoeba cells (5 × 10^5^ cells/dish) were seeded in serum- and antibiotic-free DMEM and incubated at 28°C. After *A. castellanii* attachment for 2 hours, *P. aeruginosa* (2.5 × 10^7^ CFU/dish) was added to the dishes with serum- and antibiotic-free DMEM. Dynamic images of adhesive *P. aeruginosa* were captured using an Olympus MV PLAPO 2XC dissection microscope. For fluorescence images, amoebae and bacteria were fixed with 2% paraformaldehyde and then stained with Fluoroshield containing DAPI after incubation with the bacteria for 10 minutes.

To assess the adhesion of treated bacteria to SIRC cells, *P. aeruginosa* was incubated with or without amoeba-soluble antigens (50 µg/mL) for 3 hours. After this incubation period, *P. aeruginosa* was centrifuged and then resuspended in serum- and antibiotic-free DMEM. After *P. aeruginosa* was added to corneal monolayers for 30 minutes, the culture medium was removed and SIRC cells were lysed. Then, to quantify the adhesion of the bacteria, we plated *P. aeruginosa* on LB agar plates at appropriate dilutions.

### Bacterial growth

To evaluate the effect of amoeba-soluble antigens on bacterial growth, overnight cultures of *P. aeruginosa* were diluted in LB medium (1:100) with amoeba-soluble antigens (50 µg/mL) or the same volume of phosphate-buffered saline in bacterial culture tubes. The tubes were incubated on a rotary shaker (170 rpm) at 37°C. Growth curves were generated in an Ultrospec 10 Cell Density Meter (Biochrom, USA). The OD_600_ was measured every 1 hour for 9 hours. A colony-forming unit assay was performed at 3 hours by generating serial dilutions with phosphate-buffered saline, which were cultured on LB agar plates. After overnight culture in the bacteria incubator at 37°C, visible single colonies were counted on LB agar plates.

### Biofilm formation assay

To measure biofilm mass, overnight cultures of *P. aeruginosa* were diluted in LB medium (1:100) with amoeba-soluble antigens (50 µg/mL) or the same volume phosphate-buffered saline and incubated on a rotary shaker (170 rpm) at 37°C. One hundred microliters of bacterial suspensions was transferred into the corresponding wells of 96-well flat-bottom plates (89626, Ibidi, Germany). Biofilms were grown for 6, 12, and 18 hours at 37°C. After that, the culture supernatant was discarded, the cells were washed using phosphate-buffered saline, and the attached biomass was stained with 0.1% crystal violet for 15 minutes. Subsequently, the staining buffer was removed, and the biofilm-embedded cells were washed with phosphate-buffered saline to remove unattached cells and residual crystal violet resuspended in 100 µL of 99.5%, vol/vol EtOH. After that, the optical density of the released crystal violet was measured with a microplate spectrophotometer (Thermo Fisher Scientific) at 550 nm (36).

### RNA extraction and real-time PCR

A Direct-zol TM RNA Miniprep Kit (Zymo Research) was used for RNA extraction. Bacteria were incubated with or without amoeba-soluble antigen (50 µg/mL) at 37°C for 3 or 6 hours, centrifuged at 8,000 × *g* for 5 minutes, and lysed using TRI Reagent in an appropriate volume. The mixture was mixed with ethanol (95%–100%) and subsequently transferred into a Zymo-Spin IICR column. The column was processed following the protocol for the Miniprep Kit. Finally, 45 µL of RNase-free ddH_2_O was added to the Zymo-Spin IICR column. After incubation for 1 minute at room temperature, the columns were centrifuged for 2 minutes to elute the total RNA, which was then stored at −80°C. The concentration and purity (A 260/A 280 ratio) of the total RNA were quantified using a NanoDrop TM 1000 instrument (Thermo Fisher Scientific, USA). To investigate the gene expression of *P. aeruginosa* virulence factors, reverse transcription-PCR was performed with the RevertAid First Strand cDNA Synthesis Kit (Thermo Fisher Scientific). To quantify the mRNA levels of *P. aeruginosa* virulence factors, *gyrB* (“5-GGCGTGGGTGTGGAAGTC-3”, “5-TGGTGGCGATCTTGAACTTCTT-3”), *ExoT* (“5-AATCGCCGTCCAACTGCA TGCG-3”, “5-TGTTCGCCGAGGTACTGCTC-3”), *ExoU* (“5-CCGTTGTGGTGC CGTTGAAG-3”, “5-CCAGATGTTCACCGACTCGC-3”), *ExoY* (“5-CGGATTCTA TGGCAGGGAGG-3”, “5-GCCCTTGATGCACTCGACCA-3”), and *algD* (“5-ACGAAGTGGTGGCGAGTTC-3”,”5- TGGTGTGCGGCATGAAGC-3”) primers were used. *gyrB* is a housekeeping gene for *P. aeruginosa* and was used as an internal control (37 – 39).

### Statistical analysis

All the experiments were performed as three independent experiments. The data are presented as the mean ± standard deviation. Statistical analysis was performed using two-tailed Student’s *t* test and one-way ANOVA.

## SUPPLEMENTAL MATERIAL

Figure S1 spectrum.02683-23-s0001.docx The expression of ExoT, ExoU, and ExoY in amoeba-soluble antigen-treated *P. aeruginosa*. After 6 hours of coincubation with or without amoeba-soluble antigens, the expression of ExoT, ExoU, and ExoY was measured by qPCR analysis.

ASM does not own the copyrights to Supplemental Material that may be linked to, or accessed through, an article. The authors have granted ASM a non-exclusive, world-wide license to publish the Supplemental Material files. Please contact the corresponding author directly for reuse.
