# Supplementary material for: A. castellanii and P. aeruginosa mutually exacerbate damage to corneal cells during coinfection
Source: Microbiol Spectr. 2023 Dec 14;12(1):e02683-23. doi: 10.1128/spectrum.02683-23 (PMC10783079; doi:10.1128/spectrum.02683-23)
Supplement: Figure S1 — The expression of ExoT, ExoU, and ExoY in amoeba-soluble antigen-treated P. aeruginosa. After 6 hours of coincubation with or without amoeba-soluble antigens, the expression of ExoT, ExoU, and ExoY was measured by qPCR analysis. [file spectrum.02683-23-s0001.docx]

**Supplemental Material**

**
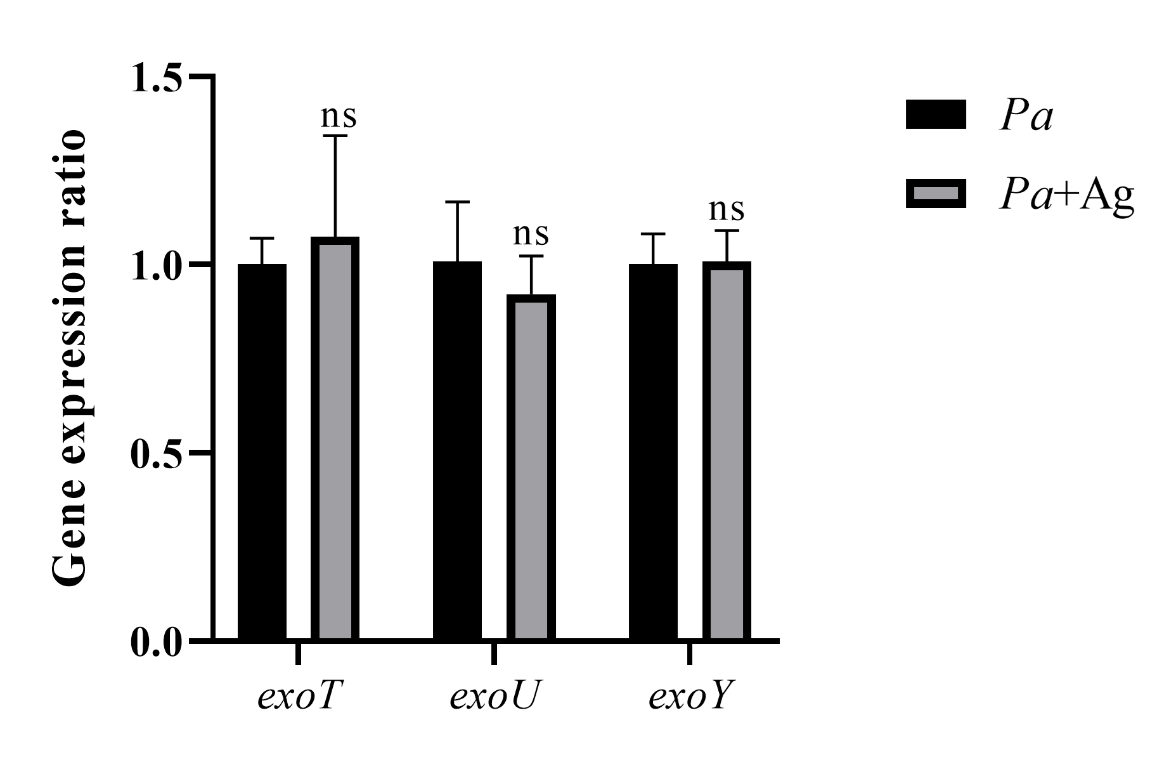
**

**Figure S1.**

The expression of *ExoT*, *ExoU*, and *ExoY* in amoeba-soluble antigen-treated *P. aeruginosa*.

After 6 hours of coincubation with or without amoeba-soluble antigens, the expression of *ExoT*, *ExoU*, and *ExoY* was measured by qPCR analysis.
